# Functional Characterization of Three Concomitant MtDNA LHON Mutations Shows No Synergistic Effect on Mitochondrial Activity

**DOI:** 10.1371/journal.pone.0146816

**Published:** 2016-01-19

**Authors:** Alberto Cruz-Bermúdez, Ramiro J. Vicente-Blanco, Rosana Hernández-Sierra, Mayte Montero, Javier Alvarez, Mar González Manrique, Alberto Blázquez, Miguel Angel Martín, Carmen Ayuso, Rafael Garesse, Miguel A. Fernández-Moreno

**Affiliations:** 1 Departamento de Bioquímica, Instituto de Investigaciones Biomédicas “Alberto Sols” UAM-CSIC and Centro de Investigación Biomédica en Red en Enfermedades Raras (CIBERER), Facultad de Medicina, Universidad Autónoma de Madrid, Madrid, Spain; 2 Instituto de Investigación Sanitaria Hospital 12 de Octubre (i+12), Madrid, Spain, and Centro de Investigacion Biomédica en Red en Enfermedades Raras (CIBERER), Madrid, Spain; 3 Departamento de Bioquímica, Biología Molecular y Fisiología, Facultad de Medicina, Universidad de Valladolid, Valladolid, Spain; 4 Hospital de Móstoles, Universidad Rey Juan Carlos, 28935, Madrid, Spain; 5 Department of Genetics, IIS-Fundacion Jimenez Diaz University Hospital (IIS-FJD, UAM), Madrid, Spain, and Centro de Investigacion Biomédica en Red en Enfermedades Raras (CIBERER), Madrid, Spain; National Eye Institute, UNITED STATES

## Abstract

The presence of more than one non-severe pathogenic mutation in the same mitochondrial DNA (mtDNA) molecule is very rare. Moreover, it is unclear whether their co-occurrence results in an additive impact on mitochondrial function relative to single mutation effects. Here we describe the first example of a mtDNA molecule harboring three Leber's hereditary optic neuropathy (LHON)-associated mutations (m.11778G>A, m.14484T>C, m.11253T>C) and the analysis of its genetic, biochemical and molecular characterization in transmitochondrial cells (cybrids). Extensive characterization of cybrid cell lines harboring either the 3 mutations or the single classic m.11778G>A and m.14484T>C mutations revealed no differences in mitochondrial function, demonstrating the absence of a synergistic effect in this model system. These molecular results are in agreement with the ophthalmological characteristics found in the triple mutant patient, which were similar to those carrying single mtDNA LHON mutations.

## Introduction

Mitochondrial complex I is the largest and least understood component of the oxidative phosphorylation (OXPHOS) system. It is the entry point for electrons into the respiratory chain and couples the transfer of electrons from NADH+H^+^ to CoQ with proton pumping across the mitochondrial intermembrane space. Complex I produces approximately 40% of the proton-motive force and is considered the major mitochondrial source of reactive oxygen species (ROS) [1].

Considerable progress has been made in recent years towards understanding the structure and function of complex I. Complex I has a characteristic L-shape structure with a hydrophobic arm embedded in the mitochondrial inner membrane and a peripheral hydrophilic arm that protrudes into the mitochondrial matrix. It can be organized in four modules according to their function: the NADH-oxidation and the quinone-reduction modules localized in the peripheral arm, and two proton-pumping modules forming the membrane arm, which are designated as proximal and distal according to the distance to the peripheral arm. The energy necessary for proton pumping is generated by the redox reactions occurring in the modules of the peripheral arm, where the flavin mononucleotide (FMN) cofactor accepts electrons from NADH+H^+^ and passes them to the CoQ reduction site, in a flow whereby electrons lose part of their energy that is used by the proton-pumping modules [2–5].

Human complex I consists of 45 subunits, of which 7 are encoded in the mitochondrial DNA (mtDNA) and the remaining 38 are encoded by the nuclear genome and later imported into mitochondria. All complex I mtDNA-encoded proteins are highly hydrophobic and are members of the proton pumping modules [6]. Mutations in any of the 45 subunits, or in the factors that assemble them, are candidates for complex I deficiencies (phenotype MIM #252010), the most common group of OXPHOS disorders in humans [7]. Mutations in mtDNA produce a remarkable spectrum of associated phenotypes, from weak single disease to devastating syndromes, due to the intrinsic nature of mitochondrial genetics and the specific energy requirements of the different cell types affected [8]. The most common mtDNA-associated complex I disease is Leber´s hereditary optic neuropathy (LHON; MIM #535000).

LHON is a maternally-inherited form of retinal ganglion cell degeneration that leads to optic atrophy and loss of central vision. LHON was the first human pathology to be associated with a mtDNA point mutation [9]. Currently, three mtDNA point mutations, the so-called three primary or common LHON mutations, affecting subunits of complex I, are described to be responsible for 95% of LHON cases: m.3460G>A, m.11778G>A and m.14484T>C [10]. Although literature on complex I deficiency and mitochondrial dysfunction induced by these primary mutations is extensive, the data is also somewhat contentious, particularly regarding the severity of such alterations. This could be in part due to the comparison of different parameters (such as mitochondrial ATP synthesis or oxygen consumption), biological sources (such as muscle biopsies, lymphoblasts, fibroblasts or hybrids, among others), and techniques utilized (such as spectrophotometry for in vitro respiratory chain complex activity and in vivo respiration, or phosphorus magnetic resonance spectroscopy (31P-MRS)) [11].

In addition to the three primary mutations, approximately 30 different point mutations have been associated with LHON and are classified into two groups; the “top-14”, which include the three primary mutations, and a group of single family or singleton cases, such as the m.11253T>C mutation described in this study.

LHON has an incomplete penetrance and commonly presents a delayed onset, which implies that the phenotypic expression may involve other factors in addition to the known mutations, such as the nuclear and mitochondrial genetic background, environmental and age-related factors [12–14].

The presence of three concomitant pathogenic mutations in a single mtDNA molecule has not been described in the literature, and therefore comparisons with similar situations are challenging. However, the presence of two non-severe mtDNA mutations in the same molecule, although also very rare, has been described in a few LHON cases. Accordingly, the co-occurrence of two of the three primary LHON mutations has been detected in five families (Table 1, row A) [15–18]. Furthermore, four studies have described the concomitant presence of one of the three common LHON mutations together with one of the remaining “top-14” LHON mutations (Table 1, row B) [19–22]. Finally, there are several cases of LHON patients (examples are shown in Table 1 row C) simultaneously harboring one of the three primary LHON mutations and a non-LHON related mtDNA mutation, including m.1555A>G, associated with aminoglycoside-induced non-syndromic hearing loss [23, 24], a common deletion related to progressive external ophthalmoplegia (PEO) and Kearns–Sayre syndrome (KSS) [25], or m.12192G>A, associated with cardiomyopathy [26].

**Table 1:** Synergistic effect of concomitant mtDNA mutations with at least one LHON-associated mutation. All mutations and their classifications are described in MITOMAP [10].

Mutations^a^			Heteroplasmy (%)	Synergy	Evidence	Functional studies	Reference
***A***							
m.11778G>A	m.3460G>A	-	100 / 10	Suggested	clinical	No	[17]
m.11778G>A	m.14484T>C	-	(23–77)^b^/ 100	No	clinical	No	[16]
m.11778G>A	m.14484T>C	-	(94/31)^c^/ 100	Yes	biochemical	Yes^d^	[15]
m.11778G>A	m.14484T>C	-	100/ (70/30)^c^	ND^e^	ND	No	[12]
***B***							
m.11778G>A	m.14502T>C	-	ND	Suggested	clinical	No	[22]
m.11778G>A	m.14502T>C	-	100 / 100	Suggested	ND	No	[21]
m.11778G>A	m.14502T>C	-	100 / 100	Suggested	clinical	No	[19]
m.11778G>A	m.14484T>C	-	(100 / 100)	Suggested	clinical	No	[20]
***C***							
m.11778G>A	m.1555A>G	-	100 / 100	Suggested^f^	clinical	No	[24]
m.14484T>C	m.1555A>G	-	100 / 100	Suggested^f^	clinical	No	[23]
m.11778G>A	common deletion	-	100 / (0–62)^g^	NO	clinical	No	[25]
m.11778G>A	m.12192G>A	-	100 / 100	Suggested^f^	clinical	No	[26]
***D***							
m.11778G>A	m.14484T>C	m.11253T>C	100/100/100	NO	biochemical	Yes	This work

a) In all rows, the first mutation belongs to the three primary LHON mutations (>95% of LHON cases). Row A, the second mutation also belongs to the three primary LHON mutations. Row B, the second mutation belongs to the remaining “top-14” primary LHON mutations. Row C, some of the cases in which the second mtDNA mutation is not described to be associated with LHON but instead to other mitochondrial diseases, such as aminoglycoside-induced non-syndromic hearing loss (m.1555A>G), progressive external ophthalmoplegia (PEO) and Kearns–Sayre syndrome (KSS) (≈ 5kb common deletion) and cardiomyopathy (m.12192G>A). Row D, the triple LHON mutation described in this study; two mutations belong to the three primary LHON mutations and the third is associated with LHON in a single family (see text).

b) Range in two families analyzed.

c) Proband/asymptomatic mother of the proband

d) Complex I dependent respiration and complex I *in vitro* activity

e) ND: no data or authors do not comment.

f) Affecting only LHON, not hearing loss, phenotype. Proband did not present hearing loss symptoms

g) Common deletion is absent in mtDNA from blood, 58% in orbicularis oculi muscle and 62% in quadriceps femoris muscle

As shown in Table 1, 7 out of 9 of these studies suggest a possible synergistic effect of the mutations strictly based on the clinical penetrance and a possible involvement of the nuclear background. The two remaining studies suggest that there are no synergistic effects between LHON mutations, based only on clinical observations. None of the studies carried out a complete biochemical analysis and only one [15] performed biochemical assays to measure complex I-linked respiration and *in vitro* activity. Nevertheless, this limited analysis was sufficient to indicate a synergistic effect between m.11778G>A and m.14484T>C mutations.

Although a recent extensive analysis of pathogenic LHON mutations [27] did not strictly describe LHON cases based on double mutations, the authors established a putative relationship between LHON mutations belonging to the “top-14”group (excluding the three common mutations), a series of presumed polymorphic variants and haplogroups of the mtDNA molecules. Indeed, the authors discussed a potential synergistic role of some polymorphisms on primary mutations based on their conservation patterns. However, no functional analyses were carried out to establish this.

Here we report a comprehensive molecular characterization of a homoplasmic triple LHON mutation in comparison with single primary mutations and haplogroup-specific controls in a nucleus and environment independent manner. We generated mutant transmitochondrial cybrid cell lines harboring the m.11778G>A (ND4, J1c haplogroup) and m.14484T>C (ND6, Uk1) single mutations and the concomitant m.11778G>A / m.14484T>C / m.11253T>C triple mutation (ND4, ND6, ND4; TRIPLE, H). All cybrid cells were made by fusing platelets from donors with the immortalized 143B cell line depleted of mtDNA (143B ρ^0^) [28].

Our results show a robust defect in mitochondrial function for all the cybrids carrying LHON mutations, but no synergistic effect of the triple LHON case compared with cybrids harboring only one primary mutation. This is consistent with the ophthalmological characteristics found in the triple LHON patient, which are no more severe than the phenotype found in LHON families harboring a single mtDNA mutation.

## Materials and Methods

### Probands

The triple LHON proband was a 27-year-old woman who experienced slight bilateral vision loss at age 9, with papillary pallor and central scotomas in both eyes. At 22 years of age, optical coherence tomography (OCT) showed a severe loss of thickness of the papillary retinal nerve fiber layer (RNFL). In the last few years, clinical parameters (visual acuity, scotomas, RNFL, papillary pallor, refraction) stabilized, with no additional ophthalmological characteristics of classical LHON symptoms or associated neurological alterations such as headache, eye pain, weakness, or other relevant health disorders.

The patients harboring m.11778G>A and m.14484T>C mutations showed classical LHON symptoms, such as bilateral visual dysfunction, vascular tortuosity of the central retinal vessels, scotomas, and no pain on eye movement. No other relevant symptoms were found.

The work described here was carried out in accordance with the Declaration of Helsinki for experiments involving human samples http://www.wma.net/en/30publications/10policies/b3/index.html. The only human samples used in this work were blood samples for platelet purification and fusion as described. The ethics committees of the Hospital 12 de Octubre and Universidad Autónoma de Madrid approved the study. Written informed consent for research purposes was obtained from all patients.

### Cell lines and growth conditions

To perform a precise analysis of the functional effect of mtDNA mutations, we minimized the influence of the nuclear background by isolating and confirming the absence of mtDNA and mitochondrial function in single expanded osteosarcoma 143B ρ^0^ cell, which was used as a progenitor for all transmitochondrial fusions. Cybrids were generated by fusion with platelets as described elsewhere [28, 29]. Briefly, platelets were centrifugated (15°C, 1,500 x g for 15 min) from plasma and 10^6^ ρ0 cells in 2 ml of complete media were carefully added to the platelet pellet followed by centrifugation at 180 x g for 10 min. The resulting pellet was resuspended in 100 μL of 42% polyethylene glycol, incubated for 1 min, and cultured in 10 ml of complete Dulbeco´s modified Eagle´s medium (DMEM, Life Technologies) as described below. After fusion for 12 hours, cells were grown in selective medium without uridine. Individual clones were isolated by limiting dilution and mtDNA was analyzed by RFLP mapping and sequencing.

Seven different cell lines were constructed in total; three from donors harboring the individual mutations m.11778G>A (ND4, J1c), m.14484T>C (ND6, Uk1) and the triple m.11778G>A / m.14484T>C / m.11253T>C (TRIPLE, H) mutation, and four cell lines from healthy donors with the corresponding haplogroups, K (J1c), K (Uk1), and two for K (H) from different pedigrees. All experiments were performed with two independent clones of each line at least in triplicate, therefore avoiding as much as possible a putative clonal effect [29, 30]. To exclude differences due to mtDNA levels after fusion, all cell lines were evaluated for mtDNA copy number several times during experiments. No significant differences were detected after stabilization (data not shown) and all experiments were performed with the cell lines at a similar passage number.

Cells were maintained in complete DMEM supplemented with 4.5 g/l glucose, 10% FBS, 50 μg/mL uridine and antibiotics. Prior to experiments, cells were cultured for 24 h in DMEM containing 2 g/L glucose and 2.5 g/L galactose (Glu/Gal medium). Cell lines were checked routinely for mycoplasma contamination.

### Genetic analysis

Patient DNA was extracted from blood using the DNeasy Blood and Tissue Kit (Qiagen). mtDNA was amplified from total DNA in 24 overlapping PCR fragments of approximately 800 bp in length, and both strands were sequenced. mtDNA sequence analysis was carried out using the revised Cambridge Reference Sequence (rCRS) of human mitochondrial DNA (GenBank number NC_012920) as a reference. mtDNA samples were classified into mtDNA haplogroups according to their sequence [31]. No LHON-associated mutations other than those described [10] were found in the mtDNA samples.

Primary LHON mutations were analyzed by RFLP in all cybrid lines from patients and healthy donors. PCR conditions were: 30 cycles of 55°C for annealing and 15 sec of polymerization. For m.11778G>A, the forward primer (5´-TTCACCGGCGCAGTCATTCTCATAA-3´) and the reverse primer (5´-TGTTGTGGTAAATATGTAGAG-3´) generate a 317 bp product containing an internal LweI recognition site in wild-type molecules, whose digestion generates fragments of 223 bp and 94 bp. For m.14484T>C, the forward primer (5´-ATAGCCATCGCTGTAGTATATCCAAAGACAACGA-3´) and the reverse primer (5´-CCGTGCGAGAATAATGATGTATGC-3´) generate a 236 bp product including a MboI restriction site in wild-type molecules, which generates fragments of 202 bp and 34 bp. All RFLP assays were carried out with positive and negative controls. Diagnostic restriction endonuclease digestions were resolved by 2% or 3% agarose gel electrophoresis for the m.11778G>A and the m.14484T>C, respectively. The m.11253T>C mutation was analyzed by sequencing the 271 bp mtDNA fragment obtained by PCR using as forward primer 5´-CCACACTTATCCCCACCTTG-3´ and 5´-AAGTGGAGTCCGTAAAGAGGTATC-3´ as a reverse primer with the same PCR conditions as above, but with an annealing temperature of 60°C.

### Growth curves

Cell growth was measured in cells seeded in 6-well plates at an initial density of 25,000 cells/well. Growth was monitored over 4 days in DMEM containing either 4.5 g/L glucose or 0.9 g/L galactose as a carbon source. Cells were harvested and counted every 24 hours.

### Oxygen consumption

The basal respiration of intact cells was measured at 37°C in Glu/Gal DMEM using a Clark-type oxygen electrode (Hansatech Instruments) as described [32]. At least three independent recordings were obtained to generate a mean.

### Lactate production

Lactate was measured in cell culture medium following a 48-h culture period as previously described [33]. Briefly, 1x10^5^ cells were plated per well in a 6-well plate in Glu/Gal DMEM. After 48 h, 50 μL of the medium was removed, deproteinized and frozen until measurement. Lactate was determined with a lactate dehydrogenase-based activity assay (Roche), which results in the production of NADH and an increase in absorbance at 340 nm that is proportional to the lactate present in the samples. The concentration of lactate calculated with a standard curve was normalized to the total amount of protein in the culture dishes measured with the Micro BCA Protein Assay Kit (Thermo Scientific). Assays were performed in triplicate in three independent experiments.

### ATP steady-state measurement

Mitochondrial steady-state ATP levels were measured in cells previously incubated with 5 mM 2-deoxy-D-glucose/1 mM pyruvate using the luminometric luciferin-luciferase-based method (CLS II, Roche Applied Science) as described elsewhere [34]. Experiments were performed in duplicate on at least three independent days.

### Measurement of ROS and MIMP

Mitochondrial superoxide production was measured using MitoSOX^TM^ Red (Invitrogen). The mitochondrial inner membrane potential (MIMP) was evaluated as the ratio of tetramethyl rhodamine ester (TMRE, Invitrogen) and MitoTracker Green (MTG, Invitrogen). To perform these assays, 0.75x10^5^ cells were grown in Glu/Gal DMEM. After addition of the fluorophores (5 μM MitoSOX^TM^ Red or 100 nM TMRE and 100 nM MTG) and incubation at 37°C for 30 min in the dark, the cells were collected in Glu/Gal DMEM and analyzed immediately with a Cytomic FC500 MPL flow cytometer (Beckman Coulter). Forward and side scatter were used to gate the viable population of cells and the mean fluorescence intensity was determined with MXP software (Beckman Coulter). Experiments were performed in duplicate on at least three independent passages.

### Enzymatic activity of OXPHOS complexes assays

The activity of the OXPHOS complexes and the enzyme citrate synthase was determined as previously described [35] using a Beckman Coulter DU 800 spectrophotometer.

### Complex I assembly

Blue native-polyacrylamide gel electrophoresis (BN-PAGE) and two-dimensional BN-SDS-PAGE analysis of OXPHOS complexes was performed as previously described [36]. For two-dimensional BN-SDS-PAGE experiments, cells were grown with 10 μg/ml chloramphenicol (Sigma) for 2 days to deplete respiratory chain complexes. Cells were then collected 48 h after chloramphenicol removal. The detection of fully-assembled complexes was carried out by western blotting after BN-PAGE using anti-NDUFA9 and anti-CII 70 kDa antibodies (Invitrogen, Mitosciences).

### [Ca^2+^]_M_ measurements

The Ca^2+^-sensitive photoprotein aequorin was used to measure mitochondrial [Ca^2+^] ([Ca^2+^]_M_). Cells were plated in 13-mm round coverslips and transfected for expression of mitochondrially-targeted low-Ca^2+^-affinity aequorin (see [37] for more details). Twenty-four-hours later, cells were incubated in standard medium (145 mM NaCl, 5 mM KCl, 1 mM MgCl_2_, 1 mM CaCl_2_, 10 mM glucose and 10 mM HEPES, pH 7.4) for 1–2 h with 1 μM coelenterazine for aequorin reconstitution. Cells were then placed in the perfusion chamber of a purpose-built luminometer. For stimulation of intact cells, cells were perfused with standard medium containing an agonist (100 μM histamine). For experiments with permeabilized cells, standard medium containing 0.5 mM EGTA instead of Ca^2+^ was perfused for 1 min, followed by 1 min perfusion with intracellular medium (130 mM KCl, 10 mM NaCl, 1 mM MgCl_2_, 1 mM potassium phosphate, 0.5 mM EGTA, 5 mM L-malate, 5 mM glutamate, 20 mM Hepes pH 7) containing 100 μM digitonin. Then, intracellular medium without digitonin was perfused for 5 min, followed by perfusion with a 4.5 μM [Ca^2+^] buffer obtained using an EDTA/Ca^2+^/Mg^2+^ mixture. The temperature was set to 37°C. Calibration of the luminescence data into [Ca^2+^] was made using an algorithm adjusted to the calibration of the aequorin form used, as described [37]. Assays were performed in duplicate with at least three independent experiments.

### Statistics

The results were presented as the mean ± SD. Statistical analysis was performed using one-way ANOVA and Tukey’s multiple comparison test. A p-value <0.05 was considered as significant.

## Results

### Comparison of mtDNA molecules

Although literature on LHON mutations is extensive, it is somewhat confusing and on occasion controversial with regards to molecular and clinical consequences. Thus, complex I deficiency varies from 0% to 80% depending on the technical approach used (enzyme activity, ATP synthesis, oxygen consumption, growth on galactose, among others), the system studied (lymphoblasts, platelets, leukocytes, transmitochondrial cybrids), the genetic background (nuclear and mitochondrial) and other factors, including environmental factors [11].

To eliminate as much as possible the involvement of modifying factors, we generated transmitochondrial cybrids harboring different mtDNA mutations and haplogroup-specific controls. This, we believe, permits the most accurate comparison among mtDNA molecules. Similarly, culturing these cells under controlled conditions removes possible environmental factors. In addition, since cybridizations were performed using a common progenitor clone of 143B-ρ^0^, a cell line previously isolated and genetically and functionally confirmed, all cybrids in this study contain an identical nuclear background. To overcome the potential clonal variability during cybridization [29, 30], we analyzed at least two different clones for each cell line. Thus, the functional behaviour of all cybrid cell lines used (Table 2) is the result of different mtDNA molecules against the same nuclear background and under identical experimental conditions.

**Table 2:** Cybrid cell lines used in this study.

Haplogroup	Mutation	Cybrid line	Clone
J1c	Wt	K (J1c)	K (J1c) 1
			K (J1c) 2
	m.11778G>A	ND4 (J1c)	ND4 (J1c) 1
			ND4 (J1c) 2
Uk1	Wt	K (Uk1)	K (Uk1) 1
			K (Uk1) 2
	m.14484T>C	ND6 (Uk1)	ND6 (Uk1) 1
			ND6 (Uk1) 2
H	Wt	K (H)*	K (H) 1
			K (H) 2
			K (H) 3
			K (H) 4
	m.11778G>A m.14484T>C m.11253T>C	TRIPLE (H)	TRIPLE (H) 1
			TRIPLE (H) 2

* Clones 1 and 2 are from a different family pedigree than 3 and 4.

### Mitochondrial function

Diseases caused by mtDNA mutations present a complex and broad range of clinical and biochemical phenotypes. We therefore analyzed different features of the bioenergetic status of cybrid cell lines. Results from the mutant cell lines were compared with those obtained from their corresponding controls to assess the effect of the mutation without any modifying elements, including haplogroups.

**Fig 1:**
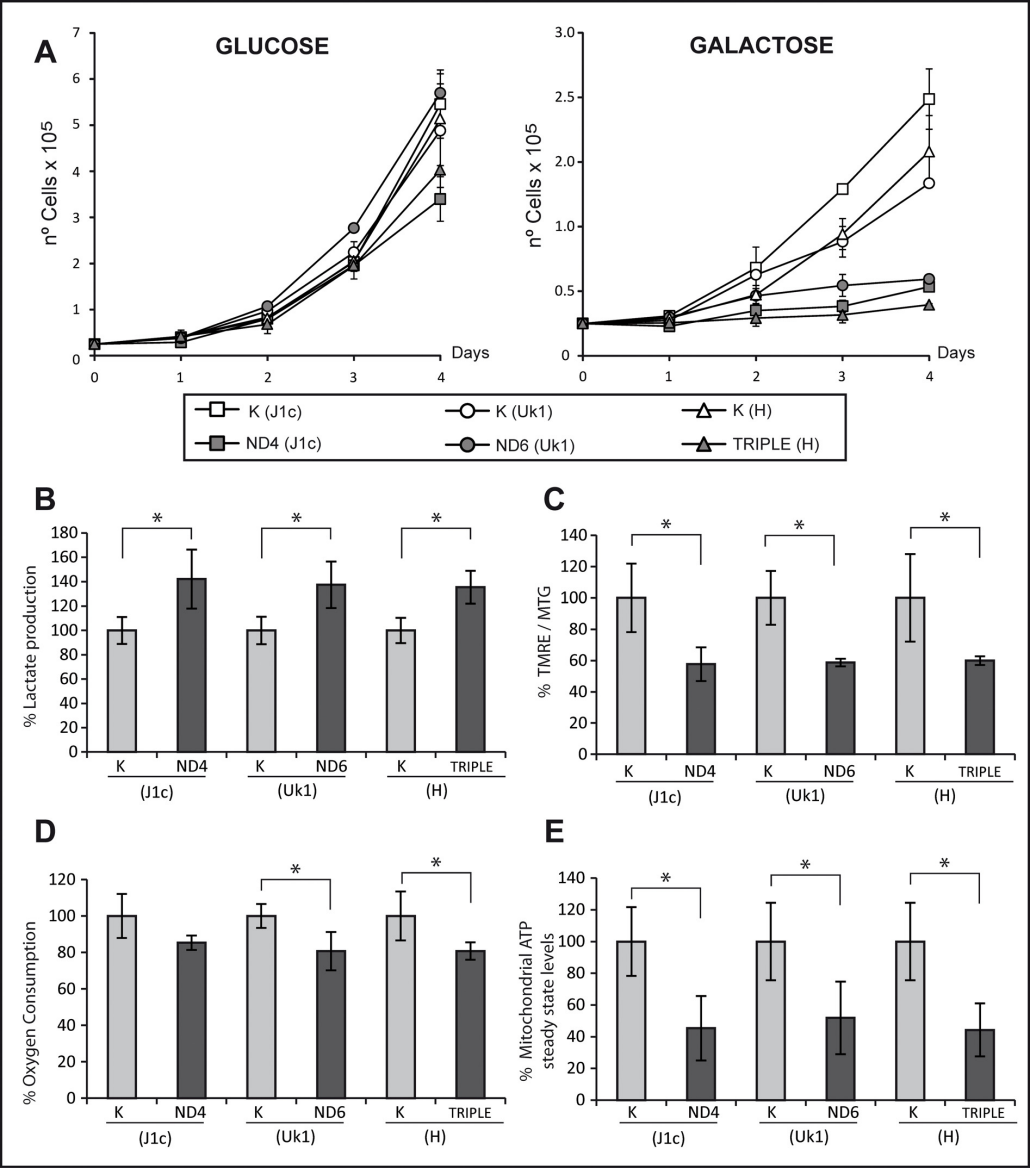
Cybrids carrying the triple mutation show similar biochemical parameters to those with single mutations. (A) Growth analysis of control and mutant cell lines. Cells were grown in glucose or galactose to evaluate their mitochondrial function. Lines represen the mean of two different clones measured in three independent experiments. Error bars represent standard deviation. (B) Lactate production was measured in culture medium following a 48-h culture period and normalized to total amount of protein. (C) Mitochondrial inner membrane potential was evaluated as the ratio of TMRE and MTG fluorescence by flow cytometry. (D) Oxygen consumption of 4×10^6^ cells was recorded for 30 minutes using a Clark-type O_2_ electrode. (E) Mitochondrial steady-state ATP was measured in cells incubated with 5 mM 2-deoxy-D-glucose and 1 mM pyruvate for 2.5 h. ATP was normalized to the total amount of protein. Bars in histograms are the mean from two different clones measured in three independent experiments. Data are represented as percentage relative to their haplogroup control. Error bars indicate standard deviation. * p<0.05 determined by one way ANOVA followed by Tukey´s test for multiple comparisons.

As a first approach, we compared their ability to grow using glucose or galactose as a carbon source. All cybrid cell lines showed comparable growth rates after 4 days in high glucose medium (Fig 1A); however, when cells were switched to galactose medium, forcing a dependence on mitochondrial function [38], the cybrid lines with LHON mutations showed a clear growth defect compared with control cells. No significant differences were found between the triple LHON cybrids and single mutants (Fig 1A).

Glycolysis and oxidative metabolism are highly regulated in the vast majority of cells. A decrease in oxidative metabolism capacity, such as that resulting from mtDNA mutation, reduces pyruvate flux into mitochondria, forcing its transformation to lactate [39]. Because lactate is balanced inside and outside of the cell by monocarboxylate transporters [40], the oxidative metabolism state can be assessed by measuring lactate levels in culture medium. Culture medium from mutant cell lines contained significantly more lactate (approximately 40%) as compared with their corresponding controls. Moreover, this increase was comparable in all mutant cybrid lines independent of the mutation (Fig 1B).

The electrical potential or charge density of the mitochondrial inner membrane is a good indicator of the correct function of the electron transport chain (ETC). To measure this, we used flow cytometry to calculate the TMRE/MTG ratio in cells. TMRE is an inner membrane potential-dependent fluorescent dye that is readily sequestered by active mitochondria. Since the signal emitted by TMRE is dependent on mitochondrial mass, MTG (a membrane potential-insensitive mitochondrial mass dye) is loaded in the same experiment to measure the charge/ mitochondrial mass relationship. A significant decrease in the TMRE/MTG ratio (approximately 40%) was detected in all mutant lines as compared with their corresponding controls. Again, no differences were detected between the mutations (Fig 1C).

To further investigate the mitochondrial molecular defects associated with LHON mutations, we next analyzed the respiratory chain capacity by measuring oxygen consumption in intact cells. Compared with their respective controls, all cybrid cell lines showed a similar and moderate decrease in oxygen consumption, which was significant for the ND6 and triple H mutations (Fig 1D). These results are consistent with those previously described in the LHON literature [13, 15, 41–43], confirming that a complex I dysfunction leads to a decrease in respiratory capacity.

ATP production is a frequently measured parameter in mitochondrial pathology and particularly in LHON studies since its synthesis is the terminal step of OXPHOS. Most studies describe a severe reduction in mitochondrial ATP production by the three common LHON mutations and, consequently, it is proposed as one of the key factors in the pathophysiology of the disease [44, 45]. As anticipated, mitochondrial steady-state ATP levels were significantly decreased by approximately 50% in all cybrid cells tested and no differences were detected between the different mutant cell lines (Fig 1E).

### Complex I performance: enzymatic activity and superoxide production

Generation of complex I deficiencies by the three primary LHON mutations is commonly accepted. However, the degree of deficiency is controversial because of the marked variability described in the literature, in part due to differences in experimental approaches. Accordingly, complex I deficiency ranges from a weak to an 80% reduction in specific activity when compared with controls [41]. This variability also depends on the mutation and only m.3460G>A (ND1) was invariably described to cause a substantial and significant decline of complex I activity [46]. By contrast, a modest decrease of enzyme activity was observed in several studies analyzing mutations in the ND6 subunit (m.14484T>C) [42] or in ND4 (m.11778G>A) [41, 47]. Remarkably, in the unique double LHON study with available biochemical data for complex I, the proband presented consistently lower complex I activity than those harboring single mutations [15].

**Fig 2:**
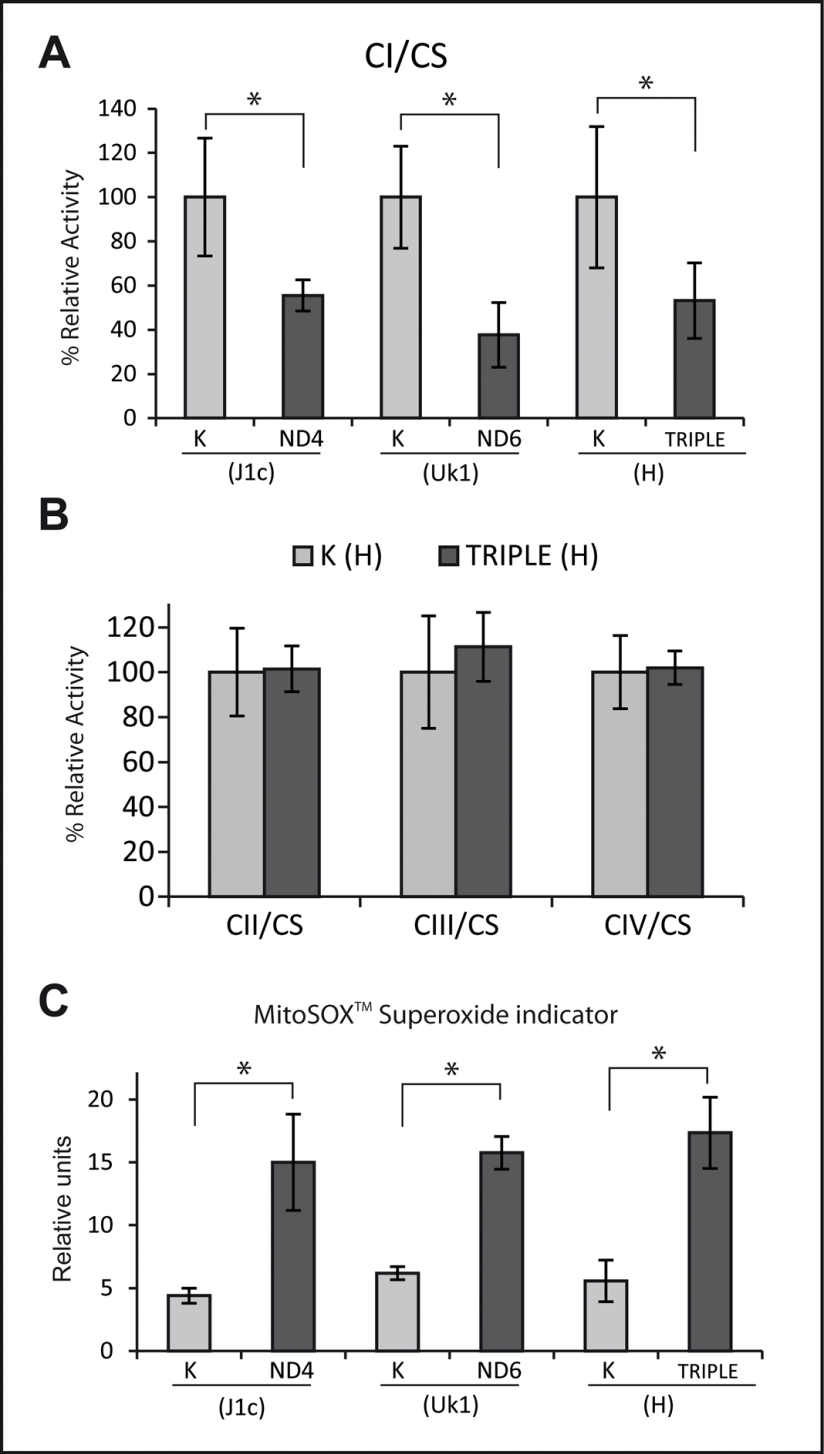
Complex I performance: mutants show the same extent of decreased complex I enzymatic activity and increased ROS production to controls. (A) Enzymatic activity of complex I was measured in all cell lines. (B) Complex II, complex III and complex IV activity in cybrids lines harboring the triple mutation and their controls from haplogroup H. In B and C, data for each cell line was normalized to the activity of citrate synthase and represented as percentage of their control haplogroup. (C) Superoxide levels were measured with MitoSOX^TM^ and flow cytometry. Data for all the experiments represent the mean of two independent clones from three experiments. Error bars are the standard deviation. * p<0.05 determined by one way ANOVA followed by Tukey´s test for multiple comparisons.

The specific activity of the respiratory chain complexes was measured in cybrid cell lines and normalized to citrate synthase activity. A significant decrease in complex I activity was detected in all mutant cybrid lines (of approximately 50%), with no significant differences between the mutations (Fig 2A). To evaluate whether the mutations were associated with an isolated complex I defect, the activity of complex II, complex III and complex IV was also measured in the triple LHON cell line and haplogroup H control cells. No differences were detected in the other OXPHOS complexes, confirming the isolated complex I defect (Fig 2B).

ROS production is generally associated with impairments of the respiratory chain. It has been hypothesized that, in addition to an ATP synthesis defect, increased ROS production may be responsible for the neurodegeneration effects underlying LHON [13]. Since impairment of electron flow through complex I leads to a higher proportion of reduced FMN that reacts with oxygen, increasing superoxide formation [48–50], we hypothesized that higher ROS levels could be present in cybrid mutants. Accordingly, mitochondrial superoxide production was evaluated with MitoSOX^TM^ and significantly increased levels of superoxide were detected in cybrid lines containing mtDNA from LHON patients as compared with their respective controls (Fig 2C). However, the differences were not significantly different between the triple LHON mutant and the single mutants. These results are in accordance with the proposed model of ROS generation and with the biochemical characterization and complex I activity, and demonstrate that the triple mutant does not generate more ROS since it has no additional mitochondrial defect.

### Complex I assembly

The importance of respiratory chain supercomplexes has been demonstrated by numerous studies in recent years [51–53], but their precise function and clinical relevance is still unclear. Nevertheless, it seems reasonable that supercomplexes would confer an advantage by physically facilitating electron transfer through the intimate association of different components of the ETC, thereby enhancing the efficiency of the process and decreasing the collateral ROS production [53]. A delayed kinetic of OXPHOS complex assembly has been postulated as a key factor in LHON pathogenesis, and the association of specific haplogroups with the primary LHON mutation is considered one of the possible modulating factors for the disease [27, 54]. In these studies, the deleterious effect of the mutations for the performance of the ETC could be exacerbated under stress conditions due to a diminished proportion of assembled supercomplexes.

**Fig 3:**
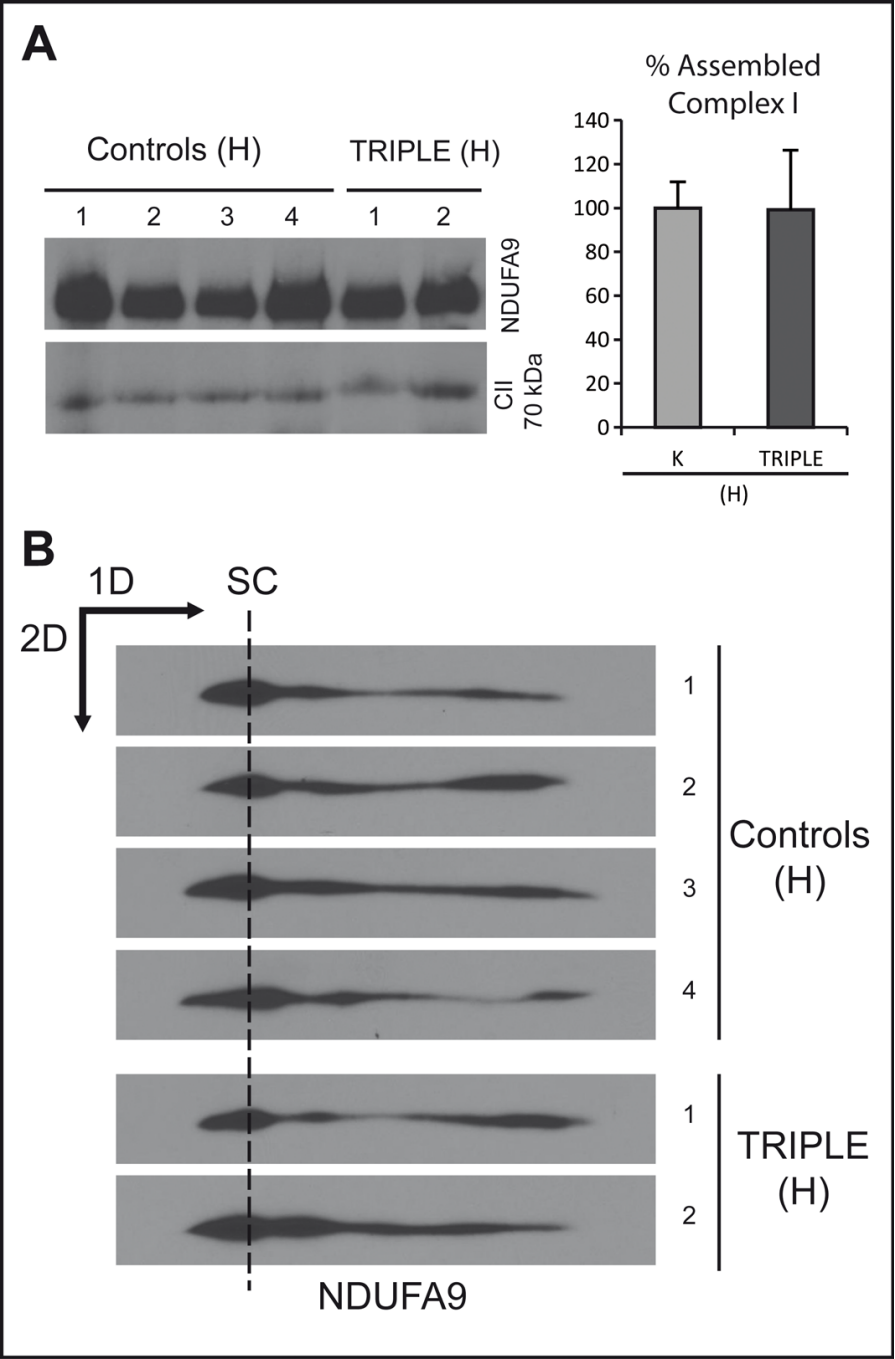
Assembly of complex I is not altered in the triple mutant. (A) Left panel: Steady-state levels of fully assembled complex I were evaluated in the triple mutant cybrid clones and in controls by BN-PAGE. Fully assembled complex I, and complex II, was determined by western blotting using antibodies recognizing NDUFA9 and CII 70 kDa, respectively. Right panel: quantification of 4 control clones and 2 triple clones from two independent gels and normalized to complex II, represented as percentage of the controls. (B) BN-SDS-PAGE of individual clones after 48 h of recovery from chloramphenicol treatment (see Materials and methods). Experiments were performed at least by duplicate. SC indicates supercomplexes containing complex I.

We analyzed the steady-state levels of complex I and the kinetics of its assembly. The former was carried out in triple LHON cybrid cells and their haplogroup controls by BN-PAGE. Complex II was used as an internal reference to account for variations in the mitochondrial mass. No significant differences were observed in the steady-state levels of fully assembled complex I in the triple mutant as compared with the wild-type haplogroup control cells (Fig 3A), as previously described for other LHON cybrids [54]. To analyze the kinetics of complex I assembly, we first depleted the OXPHOS protein complexes by chloramphenicol treatment. Analysis of subassembly patterns by two-dimensional BNSDS-PAGE of the triple LHON cybrids and controls demonstrated a similar assembly recovery (Fig 3B), strongly suggesting that triple LHON mutants do not have a complex I assembly defect, in contrast to previous results with some single mutation [54].

### [Ca^2+^]_M_ measurements

**Fig 4:**
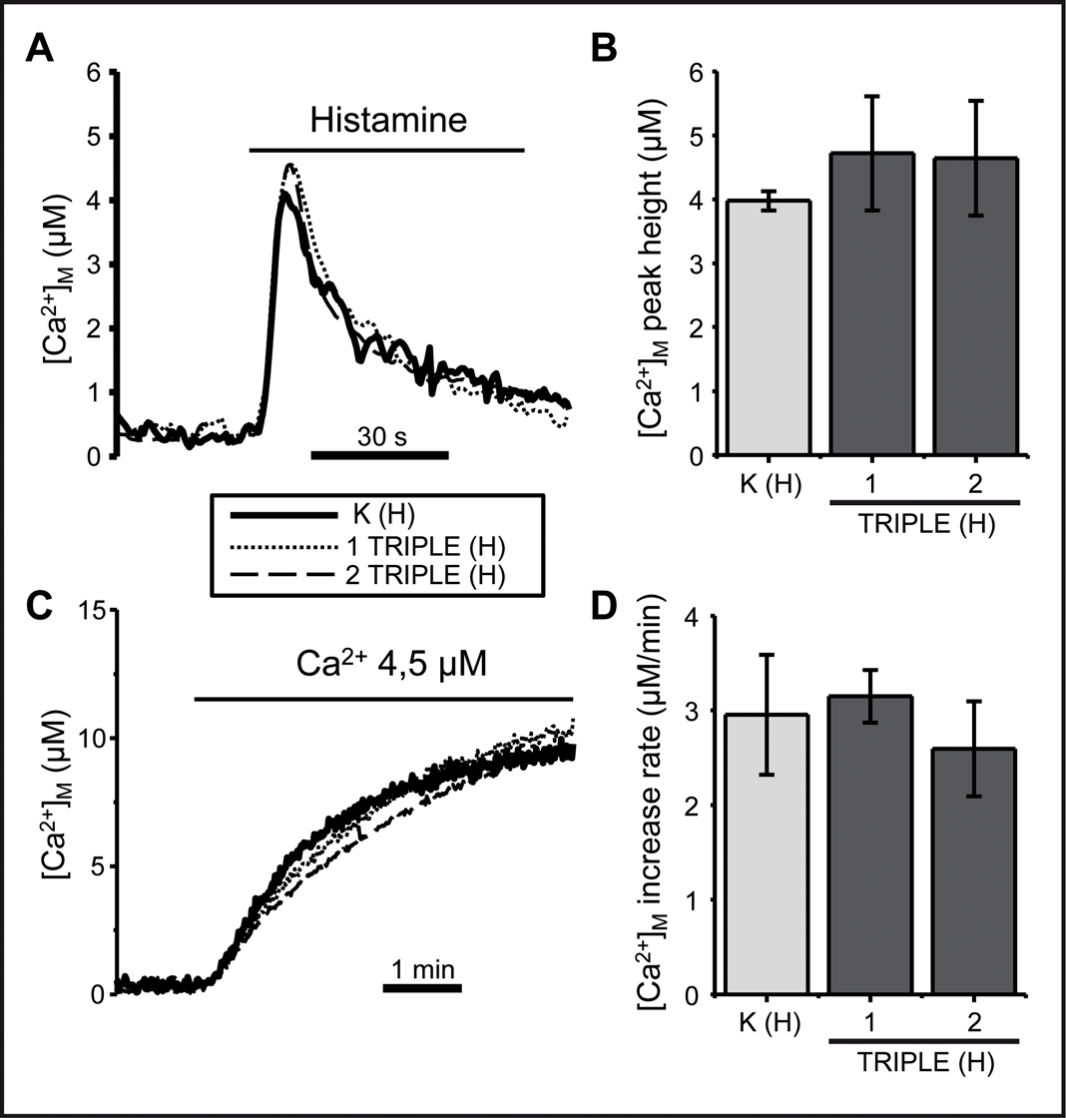
The triple LHON mutation does not affect mitochondrial [Ca^2+^] homeostasis. Mitochondrial Ca^2+^ uptake was measured under two different conditions in control cybrids and in cybrids harboring the triple mutation. (A) Intact cells were stimulated with 100 μM histamine, an agonist that induces Ca^2+^ release form the endoplasmic reticulum. The traces depict the mean of four experiments. (B) Integration of means ± SD of the peak heights (peak-basal) obtained in four measurements performed in each clone. The differences were not significant (one-way ANOVA test). (C) A 4.5 μM Ca^2+^ buffer was perfused in permeabilized cells as indicated. The traces depict the mean of three similar experiments. (D) Bars show the mean ± SD of the rates of [Ca^2+^]_M_ increase obtained in three independent experiments. No significant differences were obtained.

Mitochondrial Ca^2+^ uptake is strictly dependent on mitochondrial membrane potential and is a good parameter of mitochondrial function [55]. We measured mitochondrial Ca^2+^ uptake in triple LHON cybrids with or without permeabilzation of the cells. Mitochondrial Ca^2+^ uptake was triggered by stimulation of intact cells with histamine to release Ca^2+^ from the endoplasmic reticulum. Using permeabilized cells, mitochondrial Ca^2+^ uptake was directly induced by perfusion of a controlled [Ca^2+^] buffer. Neither agonist-induced (Fig 4A and 4B) nor perfusion-induced (Fig 4C and 4D) Ca^2+^ mitochondrial uptake was affected in the triple mutated cybrids. Since it is well documented that mitochondrial dysfunction leads to mitochondrial calcium alterations [55, 56], our results suggest that the LHON mutations described here are not sufficiently severe to alter mitochondrial Ca^2+^ homeostasis.

## Discussion

We evaluated mitochondrial dysfunction caused by the first described case described of a mtDNA molecule harboring three LHON associated mutations in comparison with two of the most common single mutations causing LHON and their corresponding haplogroup controls.

Studies of the most frequent LHON mutations in the literature indicate that these mutations are usually responsible for a subtle defect in mitochondrial function. Therefore, if the coexistence of three non-severe mutations has additive effect on mitochondrial function it should be easily detected. Moreover, because we used different clones with identical nuclei for each mutation under the same experimental conditions, our results reconcile some conflicting data in the literature regarding the impact of LHON mutations, probably as a consequence of differences in experimental methods and materials.

All functional and biochemical parameters analyzed in this study correlated well with each other and with the expected results, and revealed a defective OXPHOS system with a concomitant increase in glycolysis in all the cybrid cell lines harboring LHON mutations. The only exceptions to this were the calcium dynamics and the complex I assembly kinetics.

The involvement of mitochondria in calcium homeostasis is well documented [55, 57]. Furthermore, alterations in calcium homeostasis have been described in several severe mitochondrial pathologies [56, 58]. However, the relationship between LHON and calcium homeostasis in a cybrid model has been described in only one study [59]. Accordingly, in teratoma-derived cybrids, the m.11778G>A (ND4) mutation alters the calcium buffering ability of mitochondria. However, in our osteosarcoma-derived cybrid model, we failed to find any disarrangement in calcium homeostasis for the potentially more severe triple LHON mutation, possibly suggesting that the MIMP decrease in LHON 143B-derived cybrids is not sufficient to alter calcium import into mitochondria.

The assembly of the respiratory chain complexes is essential to maintain a proper OXPHOS function; indeed, defects in this highly regulated process are associated with different pathologies [60]. Alterations of complex I assembly have been described in LHON, suggesting a possible mechanism for the pathogenesis of the disease pathogenesis [54]. However, no defect in complex I assembly was detected in the triple mutant in the present study. We conclude from this that the mild OXPHOS impairment caused by LHON mutations was not the result of an assembly defect in complex I.

It is clear that the three simultaneous mutations do not exert an additive effect on complex I or mitochondrial dysfunction since none of the studied parameters showed a more severe defect in the triple LHON cybrids compared with cybrids carrying either m.11778G>A or m.14484T>C single mutations. A study of the isolated m.11253T> C mutation would be interesting to rule out the rare possibility of a compensating effect, unfortunately, we did not have access to patients harboring this mutation.

To our knowledge, this is the first exhaustive study on the potential synergistic effect of concomitant LHON-associated mutations. We carried out a rigorous and controlled analysis of mtDNA mutations independent of potentially confounding factors, including nuclear DNA, mtDNA haplogroup and copy number and environment or experimental conditions. Furthermore, we evaluated a large number of molecular parameters in batch experiments and all of them point to the absence of a synergistic effect among the three concomitant LHON mutations analyzed. These results are consistent with the clinical characteristics of the patient, who presents a phenotype similar to many other patients harboring single LHON mutations, including those studied in this work. The similar alteration in mitochondrial activity we observe in our cell lines when compared with the clinical and molecular disorders shown in patients and other cell systems strongly suggests a relevant role for ancillary factors in the expression of this disease [11].

A possible, but speculative, explanation for why concomitant mutations in different subunits of a multiprotein complex produce a phenotype equivalent to that observed with single mutations involves the structure/function relationship and interactions of subunits. Notwithstanding the different proposed functions of ND4 and ND6, the m.11778G>A (ND4) and m.14484T>C (ND6) mutations affect complex I function in an indistinguishable molecular manner and produce the same phenotype in patients, LHON. The simplest interpretation would be to consider that although different single mutations alter the interactions between complex I subunits, the final functional outcome is the same. Concomitant mutations would result in a disruption in proton and electron flow similar to that observed with the more deleterious single mutation, and would be a limiting factor. Since the single LHON mutations analyzed caused similar complex I defects, perhaps through a common pathogenic mechanism, an additive effect might not be produced when they coexist.

Nevertheless, it is difficult to make a theoretical prediction about putative synergistic effects of co-occurring mutations in the same complex, particularly if many structural and functional aspects of that complex are still unknown, which is the case for respiratory chain complex I. Moreover, the discrepancies in the literature on LHON patients, and different cellular models used, cannot exclude the involvement of particular forms of nuclear subunits, which could modulate, positively or negatively, the integration of all nuclear and mitochondrial subunits and, consequently, the molecular and clinical phenotype. Undoubtedly, much more work is still necessary to unravel the complex relationships among concomitant mutations in mtDNA.

## Abbreviations

MIMP: Mitochondrial Inner Membrane Potential
FCCP: Carbonyl cyanide-4-(trifluoromethoxy)phenylhydrazone)
OXPHOS: Oxidative Phosphorylation
ROS: Reactive Oxygen Species
mtDNA: mitochondrial DNA
